# Study on the spatiotemporal variation and driving factors of soil erosion based on sampling method in the loess hilly area of western Henan Province, China

**DOI:** 10.1371/journal.pone.0338185

**Published:** 2025-12-05

**Authors:** Zhijia Gu, Keke Ji, Gaohan Xu, Ao Li, Yi Shen, Chong Yao

**Affiliations:** 1 School of Geographical Sciences, Xinyang Normal University, Xinyang, China; 2 North–South Transitional Zone Typical Vegetation Phenology Observation and Research Station of Henan Province, Xinyang, China; 3 Faculty of Geographical Science, Beijing Normal University, Beijing, China; Escola de Engenharia de São Carlos da Universidade de São Paulo: Universidade de Sao Paulo Escola de Engenharia de Sao Carlos, BRAZIL

## Abstract

The loess hilly area of western Henan Province is one of the most serious areas of soil erosion due to rugged terrain, steep slopes and weak soil resistance ability. The prevention and control of soil erosion needs to know the rate, area and distribution of soil erosion in the region, so as to accurately plan and place the corresponding soil and water conservation measures. However, the study of temporal and spatial pattern, evolution and driving force of soil erosion in this region are far more enough. Therefore, this study conducted a quantitative evaluation of soil erosion in 2011 and 2022 in the loess hilly area of western Henan Province through sampling method and field investigation based on the Chinese Soil Loss Equation (CSLE). The spatial and temporal variation of soil erosion and the driving forces of soil erosion evolution were analyzed by using geographic detector method to reveal the key driving factors affecting soil erosion. The results showed that soil erosion in the loess hilly area of western Henan showed a “double decline” (decline of soil erosion rate and soil erosion area) trend in 2022. Compared with 2011, the average soil erosion rate of the investigation units was reduced by 25.5%, and the percentage of soil erosion area was reduced by 34.0%. In 2011, the areas with high soil erosion rates were mainly distributed in the southeast of Yiyang County, the southwest of Yichuan County, the north of Song County and the southeast of Luoning County. The distribution of high value areas was scattered, mainly in the west of Shangjie District, Xingyang City and Jiyuan City. Soil erosion mainly existed in forest land and cultivated land, followed by construction land, orchard land and grassland. High soil erosion rates were distributed in the area above 25° slope, and large percentage of soil erosion area was distributed at the slope range below 6° slope. The number and density of all land patches, except orchard land, increased significantly from 2011 to 2022. The results of geographical detector analysis indicated that population was the main factor affecting the change of percentage of soil erosion area. Shannon Diversity Index, GDP, and CONTAG were identified as the key factors influencing the distribution and variation of soil erosion rates.

## 1. Introduction

Soil erosion has become a serious environmental issue for human environment, global ecology, natural resources, and socioeconomic development [1,2]. Soil erosion is a major environmental problem in China. In 2023, the total soil erosion area was 2.6276 million square kilometers. The area influenced by water erosion encompassed 1.0714 million square kilometers, representing 40.77% of the overall soil erosion area [3]. Despite considerable government efforts to control soil erosion, the Loess Plateau remains one of the most eroded areas worldwide [4,5]. The loess hilly area of western Henan Province, belongs to the loess hilly and gully region, is located in the southeastern edge of the Loess Plateau. As the area with the most severe soil erosion in Henan Province, due to its rugged terrain, steep slopes, loose and thick loess, it is also crucial for water and soil conservation and ecological construction [6]. The loess hilly area of western Henan Province is a national key control and prevention area of soil erosion. To control soil erosion, the rate, area, and distribution of regional soil erosion must be determined to accurately plan and implement corresponding soil conservation measures. Therefore, soil erosion assessment is crucial for sustainable soil governance and decision–making [7].

As a region with severe soil erosion in Henan Province, the loess hilly area of western Henan has been studied by scholars for its soil erosion problems. Agricultural activities in the region primarily involve cultivating terraces on hilly and mountainous land, growing crops such as corn (*Zea mays*), peanuts (*Arachis hypogaea*), wheat (*Triticum aestivum*), sweet potatoes (*Ipomoea batatas*), and sesame (*Sesamum indicum*) [8]. Du et al (2021) used semivariance function and spatial autocorrelation to analyze the spatial differentiation characteristics of land use change and landscape ecological risk in the loess hilly area of western Henan [9]. The results showed that the overall landscape ecological risk showed an upward trend from 2000 to 2015. Yi et al. (2016) applied 3S technology to analyze the spatial distribution characteristics of erosion gullies [6]. The erosion phenomenon was severe in this area, and the largest eroded area was in the soil and rock mountain areas, such as Lushi County, Lingbao City, and Ruyang County. Soil erosion was serious in the loess hilly area, relatively slight in the bedrock mountain and terrace plain, and moderate soil erosion in the red soil hilly area [10,11]. Based on the analysis of the natural and geographical conditions, Zhu (2001) studied the characteristics of soil loss in loess hilly area of western Henan Province, analyzed the causes of soil loss and the harm to the sustainable development of society and economy, and then put forward specific countermeasures for soil and water conservation [12]. The effective measures of soil conservation are to change slope land into terrace, build water conservation forest, develop economic forest, close hillsides to facilitate afforestation, build silting dam and small soil and water conservation projects [13]. The existed studies on soil erosion mainly focused on the characteristics, distribution, types and effects of soil erosion. There are few studies on the spatial and temporal evolution of soil erosion in the loess hilly area of western Henan Province based on the quantitative analysis of long time series, and the driving force analysis of soil erosion is also lacking. In order to further implement measures in soil and water conservation, it is of great significance to quantify the substantial contributions to soil erosion by different driving factors as well as their interactions [14]. Therefore, investigating the distribution patterns of soil erosion and its influencing factors help formulating comprehensive measures for reducing soil erosion [15,16].

Soil erosion survey and assessment are the first step of soil conservation planning. Soil erosion assessment has been a challenging research topic due to its heterogeneous characteristics of the geomorphic process of soil erosion on the earth’s surface [17]. The grid–based survey and sampling–based survey are the two main types of soil erosion survey approaches at the regional scale [18]. For the grid–based survey, the whole region is divided into grids. The grid size was usually based on the spatial resolution of available data. Soil erosion rate of each grid is calculated by soil erosion model [19,20]. For the sampling–based survey, soil erosion is evaluated in a certain small percentage of the whole region by soil erosion model, with model parameters obtained through field surveys [21,22]. The sampling–based survey has been applied since 1950s, and the approach has been continuously improved. In China, a multi–stage, unequal probability, systematic area sampling method was first employed for the national soil erosion survey of 2011. A total of 32,948 investigation units were investigated, the survey results provided basic information for national conservation planning and policymaking. Compared with the grid–based survey, sampling–based survey allows detailed erosion factors to be obtained through field surveys, and thus can calculate accurate soil erosion rates within the investigation units. Besides, sampling–based survey has been proved its efficient and practical performance in regional soil erosion assessment [17,23].

Soil erosion is influenced by a combination of natural and human activities. Land use change has been recognized as having a significant influence on soil erosion, with the global expansion of cropland over the past century contributing to a considerable increase in soil erosion [24,25]. In recent years, the “Grain for Green” project, the key soil erosion control project and the slope land control measures have played an important role in soil erosion conservation. These measures directly change soil surface conditions and affect soil erosion rate and area. However, the study of temporal and spatial pattern, evolution and driving force of soil erosion in this region is far more enough. Therefore, this study conducted a quantitative evaluation of soil erosion in 2011 and 2022 through sampling and field investigation based on the Chinese Soil Loss Equation (CSLE) [26]. The CSLE, an improved version of Universal Soil Loss Equation (USLE) [27], was developed for quantitatively evaluating soil erosion considering the characteristics of landform and soil and water conservation measures in China. The purposes of this study were to quantitatively evaluate soil erosion and detect the spatiotemporal variation of soil erosion. The driving force of soil erosion evolution was analyzed by using the method of geographic detector, and the key driving factors affecting the spatiotemporal evolution of soil erosion in this region were revealed.

## 2. Materials and methods

### 2.1. Study region

**Fig 1:**
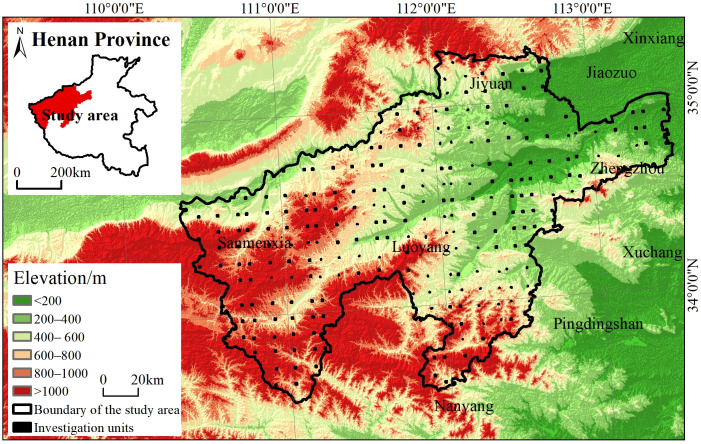
Location of the study area. The labels such as “Luoyang” and “Nanyang” on the map indicate cities surrounding the study area.

The loess hilly area of western Henan Province (33°33′–35°16′ N, 110°21′–113°30′ E) is located in the northwest of Henan Province, south of the Yellow River, between the Rocky Mountains of western Henan and the Taihang Mountains (Fig 1). The study area covers approximately 27,100 km^2^ including 19 counties and cities such as Zhengzhou, Jiyuan, Luoyang and Sanmenxia, accounting for 16.23% of the total area of Henan Province. It is situated in the transition zone between the Loess Plateau and the Huang–Huai–Hai Plain, where severe soil erosion is prevalent. The terrain is rugged with high gully density reaching 1–3 km/km^2^. Gullies occupy approximately 5%–15% of the total area, while slopes exceeding 45°, account for 60% of the territory [12]. The landform is mainly low mountains, hills and plains. The overall terrain is high in the southwest and low in the northeast, with woodland and cultivated land widely distributed. It has a temperate continental climate, located in a semi–humid zone with warm and humid summers and cold and dry winters. The territory is rich in mineral resources, coal, bauxite, molybdenum and so on, is the main coal area in Henan Province.

### 2.2. Soil erosion survey and quantitative assessment

#### 2.2.1. Data source.

The soil erosion data of 256 investigation units in the loess hilly area of western Henan in 2011 was sourced from the results of the first national water conservancy survey in 2011 [22]. The data mainly includes the position, spatial distribution, vector boundary, DEM generated by 1:10,000 contour lines of 256 investigation units, soil erosion factors of CSLE model in the investigation units, soil erosion rates and the percentage of soil erosion area. It also includes land use type, soil and water conservation practices, vegetation coverage, canopy and undercover of trees in the investigation units. The data used to calculate soil erosion of 256 investigation units in 2022 are shown in Table 1.

**Table 1:** The data used to calculate soil erosion of 256 investigation units in 2022.

CSLE factor	Input variable	Year of data collection	Source
R	Daily rainfall data	1991-2020	Monitoring results of soil and water loss in Henan Province in 2022
K	Soil properties	2022	Monitoring results of soil and water loss in Henan Province in 2022
L	1:10,000 contour lines	2010	First national water conservancy survey [22]
S	1:10,000 contour lines	2010	First national water conservancy survey
B	Land use, vegetation cover, canopy cover of forest and understory cover	2022	Field investigation
E	Engineering practices	2022	Field investigation
T	Tillage practices	2022	Field investigation

The Soil Organic Matter (SOM), Normalized Difference Vegetation Index (NDVI) and socio–economic data were derived from Data Center for Resources and Environmental Sciences, Chinese Academy of Sciences (https://www.resdc.cn/). Spatial distribution data for Gross Product (GDP) and population (POP) of 2010 and 2019 (1–km grid) were substituted for the 2011 and 2022 data, respectively. The maps of the study area were sourced from Natural Earth (public domain): http://www.naturalearthdata.com/. The methodologies for acquiring and calculating the soil erosion impact factors, as well as the model factors within the CSLE, remained consistent between 2022 and 2011, ensuring comparability of the data.

#### 2.2.2. Sample and field survey.

**Fig 2:**
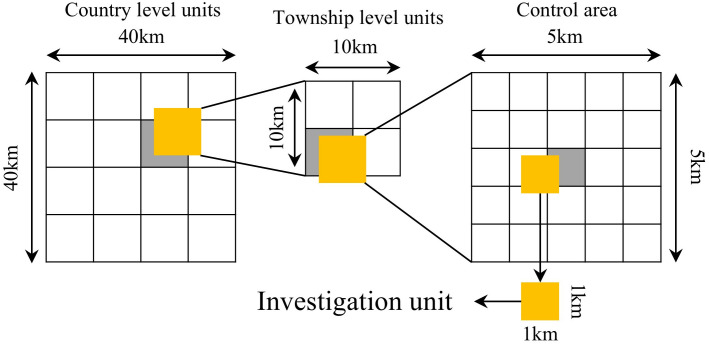
Schematic diagram of sampling survey method.

A multi–stage, unequal probability, systematic area sampling method was employed for the first national water conservancy survey in 2011 [28]. One small catchment of 0.2–3 km^2^ or a square of 1 km^2^ was sampled within each square of 100 km^2^. The schematic diagram of the selection of the field investigation units is shown in Fig 2. A total of 256 field investigation units were finalized across the entire study area. The field investigation data was transformed into grids of 10 m by 10 m within each investigation unit. The data of vegetation cover, land use type and soil and water conservation measures used in the soil erosion model factor of the investigation unit were all obtained from field investigation.

The steps of field investigation refer to the investigation process of the first national water conservancy survey, which mainly includes: taking identification photos, taking landscape photos of the plot, drawing the boundary of the plot, and filling out the water erosion field survey form. The survey scope of vegetation coverage includes typical forest land (main types of forest land in the study area), grassland and orchard land. It is necessary to investigate not only the status of cover under forest, but also the size of canopy.

#### 2.2.3. CSLE model.

Soil erosion rate was calculated using CSLE model, which was sourced from USLE [27] and developed by Liu et al (2002) [26]. All the CLSE factors have been calibrated using more than 2000 plot-years runoff data observed in China [22]. It has been well verified in the application in different regions of China [23, 29–32]. The CSLE has also been successfully applied in existing studies conducted in regions with topography and climate similar to those of the study area [29,30]. Compared with other soil erosion assessment models, it introduces surface vegetation cover and biological measures factors, engineering measures factors and tillage measures factors that are closer to the actual situation in China. The equation is as follows:


A = R × K × L × S × B × E × T (1)

where *A* represents soil erosion rate, defined as the annual average soil loss (t·ha^–1^·a^–1^); *R* represents the rainfall erosivity in MJ mm·ha^–1^·h^–1^·a^–1^, reflecting the degree of soil erosion caused by rainfall; while *K* represents the soil erodibility in t·h·MJ^–1^·mm^–1^; *L* is the dimensionless factor of slope length; *S* is the dimensionless factor of slope steepness; *B* is the dimensionless factor of biomass–control; *E* is the dimensionless factor of engineering–control; *T* is the dimensionless factor of tillage practices. The model has the capability to comprehensively forecast the soil erosion process in different regions, effectively matching the specific type and current state of soil and water conservation measures in China. Furthermore, it can accurately response the mean annual soil loss on slopes. The rainfall erosivity was calculated using daily rainfall data according to the method proposed by Zhang et al. (2002) [33] and was extrapolated to the whole study area through the kriging approach. Based on the composition of particle size and soil organic matter of soil samples of each soil type in the study area, soil erodibility was calculated using Williams model [27] and modified by observed data from runoff plot data. The factors of slope length and steepness was calculated by “calculation tool of topographic factors” based on the 1:10,000 digital contour map at investigation unit [34]. B, E and T factor were estimated depending on different land use, engineering and tillage practices of the investigation units.

#### 2.2.4. Geographical detector.

Geographic data has two attributes, one is spatial autocorrelation and the other is spatial heterogeneity (the phenomenon of intra−layer variance being less than inter–layer variance [35]. The geographic detector is an innovative statistical theory and methodology designed to assess spatial heterogeneity and examine its characteristics. It was originally used to study the mechanism by which local factors affect disease risk [36]. The method does not consider linearity and makes no linear assumptions about variables, so it is more intuitive, faster, and more efficient to measure the contribution of each factor. The geographic detector’s factor detector is utilized for identifying the spatial variation of the dependent variable *Y*, and the contribution capacity about each variable *X* to the degree of influence on the *Y* can also be obtained. The measurement of its influence degree is determined by the *q* value, with a higher *q* value indicating a greater capacity of *X* to influence the degree of variable *Y*. The formula is detailed below:


$$q=1-\frac{\text{1}}{NS^{2}}\sum {\mathrm{\backslash nolimits}}_{h=1}^{L_{h}}N_{h}S_{h}^{2}$$ (2)

where *q* is the spatial heterogeneity of an index, *q*∈[0,1]; *h* indicates the partition, *L* indicates the number of partitions, *h* = 1, 2,...; *Lh*, *N* and *N*_*h*_ represent the sample numbers of the whole region and subregion *h*, respectively; *S*^*2*^ and S^*2*^_*h*_ is the variance of the whole area and the subarea *h*; *NS*^*2*^ And $\sum {\mathrm{\backslash nolimits}}_{h=1}^{L_{h}}N_{h}S_{h}^{2}$ represent the total variance of the entire region and the sum of the variance within the region. If *q* ≠ 0, the formula is valid.

The interaction detector examines an interaction by comparing the *Q* values of the interaction with those of two individual variables (Table 2). The interaction detector investigates five different types of interactions: including nonlinear–weaken, uni–variable weaken, bi–variable enhance, independent, and nonlinear−enhance [37,38].

**Table 2:** Different forms of interaction between two covariates.

Interaction Relationship	Interaction
*Q*_*u∩v*_ < min(*Q*_*u*_,*Q*_*v*_)	Nonlinear−weaken: The interaction of two variables nonlinearly weakens the impacts of single variables.
min(*Q*_*u*_,*Q*_*v*_) ≤ *Q*_*u∩v*_ ≤ max(*Q*_*u*_,*Q*_*v*_)	Uni−variable weaken: The impacts of individual variables are weakened by the interaction, resulting in a uni–variable effect.
max(*Q*_*u*_,*Q*_*v*_) ≤ *Q*_*u∩v*_ ≤ (*Q*_*u*_,*Q*_*v*_)	Bi−variable enhance: The impact of single variables is enhanced by the interaction, resulting in a bi–variable effect.
*Q*_*u∩v*_ = (*Q*_*u*_,*Q*_*v*_)	Independent: The impacts of variables are assumed to be independent.
*Q*_*u∩v*_> (*Q*_*u*_,*Q*_*v*_)	Nonlinear−enhance: The effects of variables are enhanced in a non–linear manner.

Number of patch (NP) and patch density (PD) were selected as area indicators. Edge density (ED), mean shape index (SHAPE_MN) and perimeter area fractal dimension (PAFRAC) were selected as edge−shape indexes. Agglomeration index (AI) and Contagion index (CONTAG) were selected as the index of agglomeration and dispersion. Shannon`s diversity index (SHDI) and Shannon`s evenness index (SHEI) were chosen as indicators of diversity. Elevation (ELE), slope (S), slope length (SL), temperature (T), precipitation (PRE) and soil organic matter (SOM) were selected as natural environment index. Gross domestic product (GDP), population (POP) and night light (Light) data were added as socio–economic factors to analyze their impact on soil erosion.

In this study, based on the GCS_WGS_1984 coordinate system and 10–meter resolution raster data of influencing factors, the zonal statistics function in ArcGIS 10.8 software was employed to extract the average values of these factors within each investigation unit. Using the “Visual Binning” tool in IBM SPSS 23 software, discrete processing of the influencing factors was conducted by rationally determining segmentation points according to data distribution histograms. Initially, all factors were discretized using uniform classification criteria and incorporated into the Geographical Detector to examine the *q*–values from factor detection. After testing multiple classification schemes, the discretization approach yielding the highest *q*–value for each factor was selected as the final classification scheme for this study. The Microsoft Excel macro tool designed for Geographical Detector was utilized to perform factor detection and interaction detection analyses, treating soil erosion area, soil erosion rate for both 2011 and 2022, and their changes over the 12–year period as dependent variables, with the discretized influencing factors as independent variables.

## 3. Results

### 3.1. Temporal and spatial distribution analysis of soil erosion

#### 3.1.1. Temporal distribution of soil erosion.

According to the Standard for Classification and Grading of Soil Erosion Rate (SL190–2007) issued by the Ministry of Water Resources, PRC, and combined with the actual distribution of soil erosion in the loess hilly area of western Henan Province, the soil erosion rate of the study area was divided into 6 grades: slight, low, moderate, high, extremely high, and severe soil erosion (Table 3). The soil erosion rates and spatiotemporal distribution in the investigation units can reflect the soil erosion status in the loess hilly area of western Henan. The sum of areas with low erosion, moderate erosion, high erosion, extremely high erosion, and severe erosion constitutes the soil erosion area.

**Table 3:** Soil erosion area and percentage of the investigation units from 2011 to 2022.

Average Soil Erosion Rate /t·ha^–1^	Grade	2011		2022	
		**Area/km** ^**2**^	**Percentage/%**	**Area/km** ^**2**^	**Percentage/%**
<2	Slight	106.27	55.90	134.88	70.90
2–25	Low	70.08	36.80	42.42	22.30
25–50	Moderate	8.74	4.60	10.37	5.40
50–80	High	2.42	1.30	1.72	0.90
80–150	Extremely high	1.67	0.90	0.71	0.40
>150	Severe	1.01	0.50	0.09	0.10

Soil erosion area and percentage in the survey units changed with different erosion grades during 2011–2022 (Table 4). The average soil erosion rates in the survey units in 2011 was 7.97 t ⋅ ha^−1^⋅ a^−1^, and the average soil erosion rate in 2022 was 5.94 t ⋅ ha^−1^ ⋅ a^−1^, which was 25.5% lower than in 2011. The percentage of soil erosion area in 2011 was 44.10%, which was reduced to 29.10% in 2022. In general, soil erosion in the loess hilly area of western Henan showed a trend of “double decline”. From 2011 to 2022, low erosion, high erosion, extremely high erosion and severe erosion all showed a decreased trend. Slight erosion showed the greatest increase in both area and percentage: the area increased by 28.61 km^2^, and its percentage increased from 55.9% to 70.9%.

**Table 4:** Transfer matrix of soil erosion rates in the investigation units from 2011 to 2022 (km^2^).

2011	2022						
	Slight	Low	Moderate	High	Extremely high	Severe	2022 transition in
**Slight**	79.05	21.49	4.65	0.75	0.28	0.06	27.22
**Low**	47.04	17.53	4.57	0.62	0.30	0.03	52.56
**Moderate**	5.69	2.13	0.70	0.16	0.05	0.01	8.04
**High**	1.42	0.58	0.27	0.11	0.03	0.00	2.310
**Extremely high**	1.07	0.40	0.11	0.06	0.02	0.00	1.65
**Severe**	0.61	0.29	0.07	0.03	0.02	0.00	1.01
**2011 transition out**	55.83	24.89	9.67	1.61	0.69	0.10	92.78

* The transition out refers to the area transferred to other categories of erosion grade in 2011, and the transition in refers to the area transferred from other categories of erosion grade in 2022.

The transfer matrix between 2011 and 2022 showed the transition in area of different soil erosion grades changes. In the past 12 years, the newly increased slight erosion area was 27.22 km^2^, and the area transition in low erosion was the largest. The transition in area of moderate erosion was 4.65 km^2^, and the high, extremely high and severe erosion transition in area was small. The newly increased area of low erosion is 52.56 km^2^, which is mainly converted from the slight erosion, followed by the higher amount of moderate erosion, which is 4.57 km^2^, accounting for 8.69% of the total transition in area, and the smaller amount of high, extremely high and severe erosion, which is a total of 0.95 km^2^.The newly increased area of moderate erosion is 8.04 km^2^, which is mainly transformed by slight and low erosion, while the transition in area of high, extremely high and severe erosion is still small, and the total transition in area is 0.22 km^2^. The increased area of high erosion was 2.31 km^2^, which is come from the 1.42 km^2^ slight erosion and 0.58 km^2^ low erosion, followed by the moderate erosion transferred area of 0.27 km^2^, and the newly increased area of extremely high and severe erosion was 0.04 km^2^. The increased area of extremely high erosion was 1.65 km^2^, of which 1.07 km^2^ was transferred by slight erosion and 0.27 km^2^ by moderate erosion. The new area of high erosion was 1.01 km^2^, which was mainly increased by slight and moderate erosion, followed by 0.065 km^2^, 0.025 km^2^ and 0.022 km^2^ from moderate, high and extremely high.

During the period from 2011 to 2022, 55.83 km^2^ of slight erosion has been transferred out, which is the highest area transition out of the six erosion grades. Most of it transferred to low erosion, accounting for 84.26%, followed by moderate erosion (10.19%), and high erosion (2.54%). The area transformed into extremely high and severe erosion is small, only 3.01%. 86.32% low erosion was transformed to slight erosion. Then low erosion (2.13 km^2^) was transferred to moderate erosion. In addition, the area transformed to high, extremely high and severe erosion is small, accounting for 2.34%, 1.61% and 1.18%. 9.67 km^2^ of moderate erosion was transferred out to slight (4.65 km^2^) and moderate erosion (4.57 km^2^). Then 2.8% and 1.17% were transformed into high and extremely high erosion, and 0.07 km^2^ were transformed into severe erosion. 1.61 km^2^ of high erosion was transferred into slight and low erosion, and 0.16 km^2^ was transformed into moderate erosion. The transfer area of extremely high and severe erosion accounted for 5.22%. The transition out area of extremely high erosion was 0.69 km^2^, and the percentage of the transfer area to low and slight erosion was 43.44% and 40.52%. The percentages of the moderate, high and severe erosion were 7.87%, 4.96% and 3.21%. The converted severe erosion area (0.10 km^2^) was redistributed as follows: slight erosion (57.89%), low erosion (29.47%), moderate erosion (7.37%), high erosion (3.16%), and extreme erosion (1.05%).

In general, from 2011 to 2022, the soil erosion rate in the study area remained unchanged at 97.42 km^2^, accounting for 51.22% of the total study area. The area of soil erosion rate decreased from high to low was 32.99 km^2^. Due to the large percentage of the transition from slight erosion to low erosion, the area of the transition from low to high erosion was more, accounting for 31.44%. The high erosion in the loess hilly area of western Henan was gradually reduced, and tended to low and slight erosion.

#### 3.1.2. Spatial distribution of soil erosion.

**Fig 3:**
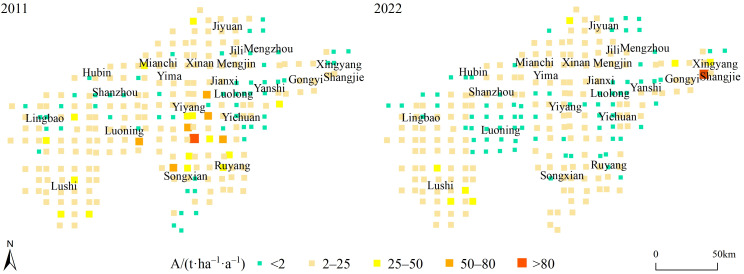
Average soil erosion rates of investigation units.

The areas with high soil erosion rates in 2011 were mainly distributed in the southeast of Yiyang, the southwest of Yichuan, the north of Song and the southeast of Luoning (Fig 3). In 2022, the soil erosion rates decreased, especially in Luoning. In terms of spatial distribution, the distribution of high–value areas tended to be scattered, mainly distributed in the west of Zhengzhou’s Shangjie, Xingyang, and Jiyuan (Fig 3). Meanwhile the soil erosion status in the southern mountainous area was improved. This is related to the development of production and construction projects in these areas in recent years. The mitigation of soil erosion in Luoning is closely related to the comprehensive soil erosion control project continuously invested in sloping farmland.

**Fig 4:**
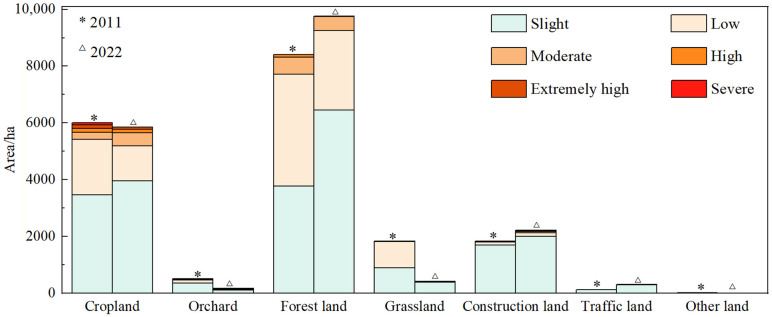
Soil erosion area of land use types.

In 2011, soil erosion mainly occurred on cropland and forest land, followed by grassland and construction land (Fig 4). In 2022, the erosion distribution of different land use types was changed, and soil erosion mainly occurred on forest land and cropland. The overall soil erosion area of forest land increased, but the low or above erosion area decreased, followed by construction land, grassland and traffic land. The soil erosion area of both cropland and grassland decreased.

**Fig 5:**
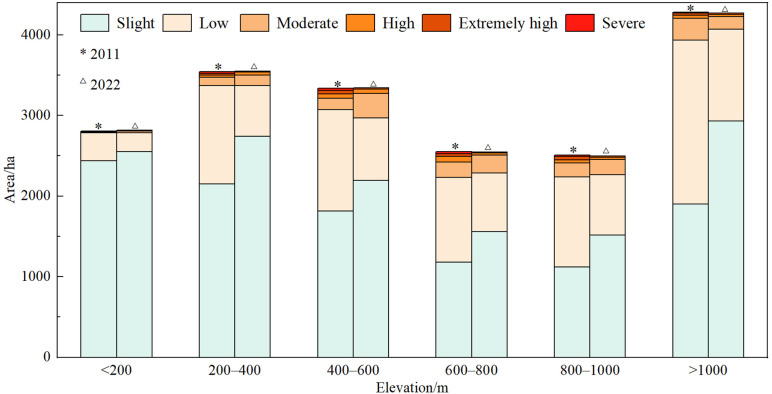
Soil erosion area at different elevations.

In 2011, low and moderate erosion were mainly concentrated in the region with altitude greater than 1000 m, and their areas accounted for 28.98% and 30.66% of the total erosion area, respectively. The high erosion was mainly distributed in area of 600–800 m above sea level, and the extremely high and severe erosion was mainly concentrated in altitudes of 400–600 m. It can be seen that soil erosion occurred in the range of 200–1000 m above sea level in 2011. In 2022, the low erosion is still concentrated in the area with the altitude greater than 1000 m, but the soil erosion rate has decreased compared with 2011, and the area has decreased from 20.36 km^2^ in 2011 to 11.38 km^2^, accounting for 26.85% of the low erosion area. Moderate erosion is mainly distributed at 400–600 m above sea level, and the altitude is lower than that of 2011, accounting for 29.21% of the total area of moderate erosion. The high erosion was concentrated at the altitude of 400–600 m, and the area was 0.55 km^2^, accounting for 31.46% of the high erosion area. Extremely high erosion was found at both 400–600 m and over 1000 m altitudes, with the latter accounting for 21.30% and 21.41% of the former. Severe erosion (0.05 km^2^) occurred mainly in the area less than 200m above sea level, accounting for 60.55% of the total severe erosion area. In 2022, soil erosion in the loess hilly area of western Henan mainly occurs at altitudes less than 200 m and 400–600 m. Compared with 2011, the altitude range of soil erosion distribution in 2022 narrowed and soil erosion became more concentrated (Fig 5).

**Fig 6:**
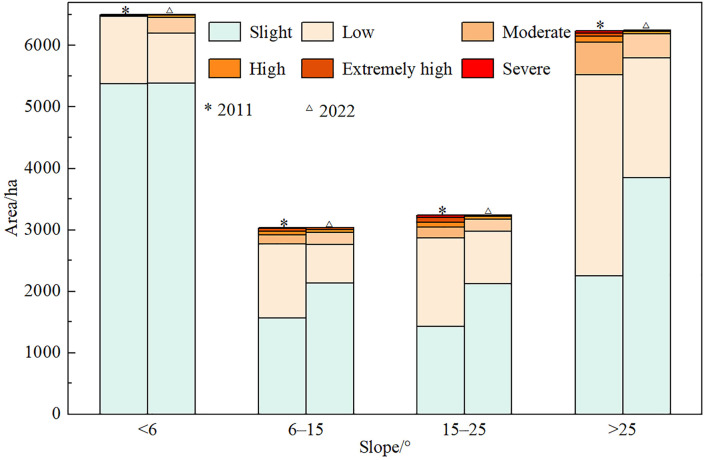
Soil erosion area of different grade of slope.

The slope of the study region was divided into four grades: < 6°, 6°–15 °, 15°–25 °, and >25°, which were superimposed with the soil erosion rate in 2011 and 2022 respectively. The area distribution of each soil erosion type in different slope ranges within the survey units was analyzed (Fig 6). In 2011, soil erosion occurred in a large area above 25°, and 47.58% of the soil erosion area was concentrated in this slope range. This was followed by 15°–25 ° and 6°–15 ° slope ranges, accounting for 21.63% and 17.45%. In 2022, the soil erosion area of all slope is decreasing, but the erosion area above 25° is still the largest, accounting for 43.38%, followed by the slope 15°–25° and below 6°, accounting for 20.24% and 20.17%. The slope range below 6° is more suitable for human activities such as agriculture and cultivation, and is subject to greater human disturbance, and the soil erosion area has little change.

### 3.2. Spatial distribution of percentage of soil erosion area

After calculating the soil erosion rates of the investigation units, if we want to get the soil erosion status covering the whole region, we need to use certain methods to expand the results of the investigation units to the regional scale. In this study, the geostatistical spatial interpolation method is used to obtain the regional soil erosion status. Taking the average soil erosion rates of the sampling unit as the interpolation index results in a large error, and can not represent the soil erosion status of the investigation units well, and loses the physical significance of the soil erosion rates. On the regional scale, especially for regional soil and water conservation planning, soil erosion rates is not a good parameter to characterize the spatial distribution of soil erosion. Therefore, we considered using the index of soil erosion area percentage in the investigation units for interpolation to obtain the assessment status of regional soil erosion [25].

**Fig 7:**
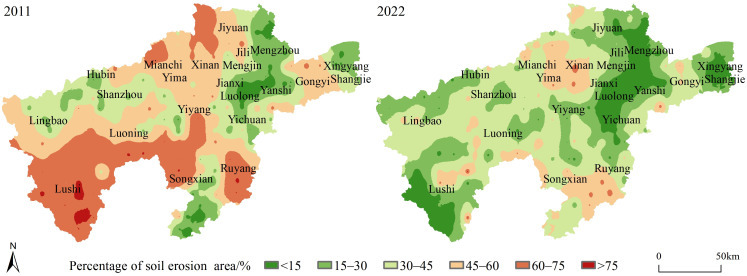
Spatial characteristics of percentage of soil erosion area from 2011 to 2022.

The percentage of soil erosion area in the study area showed an overall decreasing trend, while the local soil erosion rate increased (Fig 7). The soil erosion area accounted for less than 45%, and the area was widely distributed. In 2011, the percentage of high soil erosion area was mainly distributed in three regional ranges, namely, southwest, southeast and north of the study area. The southwest mainly includes Lu, the south of Luoning, the northwest of Song, the southeast mainly includes the central and south of Ruyang, the north mainly includes Mianchi, the north of Xin’an and the west of Jiyuan. The low value area was mainly distributed in Luoyang, the south of Song and Xingyang. Compared with 2011, the percentage of soil erosion area in 2022 was significantly reduced, and the soil erosion situation was improved. There are three low–value centers of soil erosion area percentage: Luoyang; Xingyang and Shangjie; Southwest Lu Shi. The high soil erosion area was mainly distributed in the southeast of Song and the south of Ruyang, the south and west of Xin ‘an, and the east of Yima and Mianchi (Fig 7).

During the past 12 years, the percentage of soil erosion area in the study area showed a decreasing trend, and the areas below 15% gradually increased, and the surrounding region were driven by Luoyang as the center to realize the reduction of soil erosion area. The newly added low value center in Lushi extended to Luoning, Luoyang to extended the southwest direction of Yiyang and Luoning, Xingyang has a radiation driving effect on the Shangjie, the low value area extended to the west.

### 3.3. Driving force analysis of soil erosion

#### 3.3.1. Characteristics of landscape pattern.

The landscape pattern index serves as a fundamental measure for characterizing landscapes, encompassing both the landscape structure and function [39,40]. The fragmentation of landscape refers to the process of breaking down larger patches of land cover into smaller ones, with the aim of quantitatively describing the structure of the landscape. Changes in landscape patterns and their spatial distribution patterns can significantly impact the patterns and evolution of soil erosion [41]. Based on the land use types obtained from field investigations, the landscape indices were calculated using the software Fragstats 4.2, with a spatial resolution of 10 meters for each index. NP and PD of all land use types in the study area increased significantly (p < 0.05) from 2011 to 2022, indicating that the landscape in the study area is expanding except for the orchard land, and the increase in density reflects that it is gradually being fragmented. The expansion and fragmentation of traffic land was the most obvious, followed by cropland, and the rest are construction land, forest land, grassland. The ED of orchard land and grassland decreased, indicating that the complexity of patch edge was reduced and the shape was gradually simplified. The ED index of cropland, forest land, construction land, traffic land increased. The SHAPE_MN of all regions decreased, indicating that the shape of patch was more uniform and closer to square in the basin. Except for traffic land, the PAFRAC of all land use types increased. The AI index of all land use types in the study area have decreased, indicating that the connectivity of the landscape in the area is reduced. It also reflected in the index PD and ED (Table 5).

**Table 5:** Landscape index of different land use from 2011 to 2022.

Year	Land use	NP	PD	ED	SHAPE_MN	PAFRAC	AI/%
2011	Cropland	352	1.851	30.168	1.751	1.197	96.492
	Orchard land	59	0.310	3.492	1.500	1.235	96.147
	Forest land	364	1.914	24.093	1.751	1.095	97.445
	Grass land	90	0.473	7.960	1.764	1.197	96.976
	Construction land	238	1.251	11.776	1.445	1.120	96.055
	Transportation land	25	0.132	3.619	3.430	2.179	85.976
2022	Cropland	658	3.460	40.288	1.555	1.299	95.618
	Orchard land	45	0.237	1.749	1.435	1.297	93.925
	Forest land	603	3.170	35.935	1.567	1.223	97.109
	Grass land	134	0.705	5.596	1.495	1.327	92.760
	Construction land	494	2.597	19.305	1.348	1.245	94.909
	Transportation land	642	3.376	11.447	1.280	1.570	81.550

Overall, the degree of landscape fragmentation in the loess hilly area of western Henan Province from 2011 to 2022 is relatively significant. As a result, the stability of landscape pattern is decreased, the connectivity between patches is weakened, and the heterogeneous landscapes near the boundary interact with each other, reducing the regional soil and water conservation ability [42].

#### 3.3.2. Geodetector analysis.

a) Factor detector

**Fig 8:**
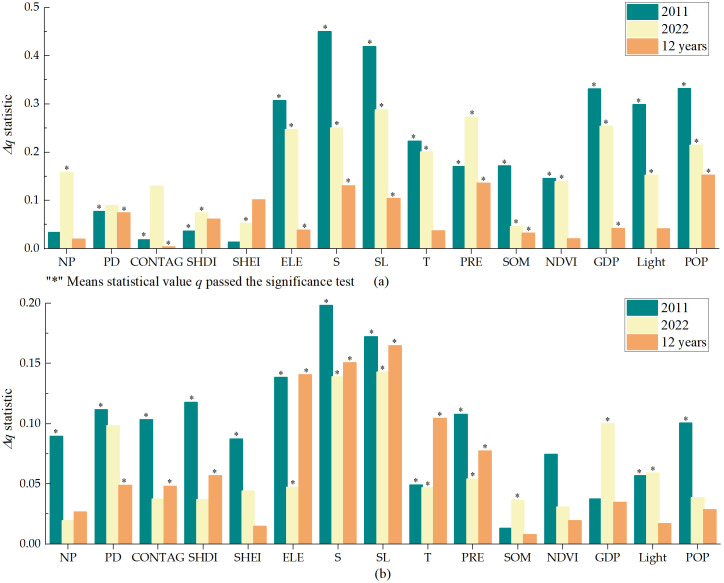
Factor geographical detection results: (a) Soil erosion area; (b) Soil erosion rate.

As shown in Fig 8 (a), the analysis results of soil erosion area percentage factor show that the dominant factors of soil erosion area in 2011 and 2022 are mainly slope and slope length, and their explanatory power to soil erosion area is the highest in the same period, with *q* values of 45% and 28.8%, but the explanatory power has decreased between 12 years. The dominant factor of area change in 12 years was POP, and *q* value was 15.1%, indicating that although the high explanatory power factor of soil erosion area size was concentrated in natural factors, the change of soil erosion area was closely related to human activities, especially population. It shows that the change of soil erosion area in the study area is based on landform and is greatly influenced by human beings. The explanatory power of diversity index (SHDI and SHEI) on soil erosion area increased, indicating that the change of soil erosion area gradually changed from the socio–economic orientation to the landscape fragmentation orientation. The landscape fragmentation degree increased within 12 years, indicating that the landscape fragmentation degree of human activities had a greater impact on soil erosion area (S1 Table).

The results of soil erosion rates factor detection are shown in Fig 8 (b). From 2011 to 2022, the dominant factor of soil erosion rates and its 12a changes in the study area is still slope length factor, with explanatory power of more than 14%, which is basically consistent with the influence factor of erosion area change. The increase of erosion area will lead to the increase of soil erosion amount to a certain extent, and the erosion rates will also increase correspondingly. In addition to natural factors, erosion area change is also related to SHDI, PD and CONTAG, with explanatory power of 5.7%, 4.9% and 4.8%. Soil erosion rate is closely related to social and economic development. People’s land planning and soil and water conservation measures greatly affect the local soil erosion rates. Rational planning of land use pattern and improvement of regional landscape pattern distribution are the main ways to effectively control soil erosion in the loess hilly region of western Henan Province.

b) Interaction Detection

**Fig 9:**
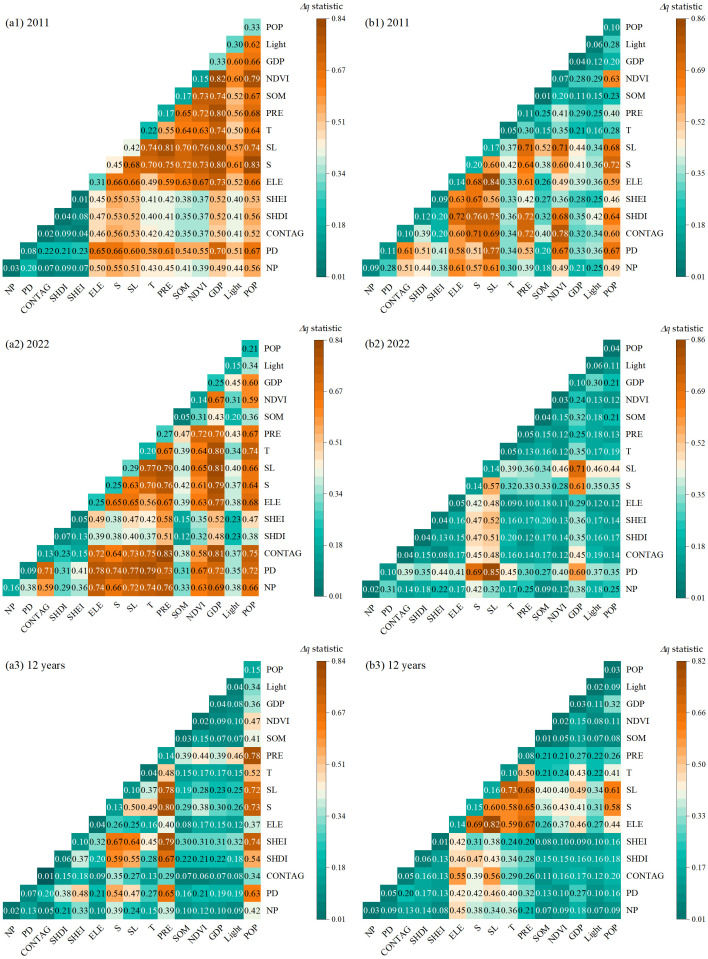
Interactive geographical detection results during 2011–2022: (a1) Soil erosion area in 2011; (a2) Soil erosion area in 2022; (a3) Changes of soil erosion area in 12 years; (b1) Soil erosion rates in 2011; (b2) Soil erosion rates in 2022; (b3) Changes of soil erosion rates in 12 years.

The results of interactive detection are shown in Fig 9. While some factors didn’t exhibit significant explanatory power individually during the factor detection test, the explanatory power was greatly improved after interacting with two factors. From the perspective of soil erosion area, the largest values of interaction in 2011, 2022 and 2012 are S∩POP, CONTAG∩PRE and S∩PRE, with *q* values of 83.5%, 83.4% and 80.2%. This indicates that the erosion area is affected by slope, as well as the change of land use pattern connectivity caused by human activities, which further changes the number of patches in different places, leading to the fragmentation of landscape pattern, and further affects the soil erosion area. The interaction of human activities, especially population, has sustained and extensive changes in soil erosion area, especially in the 12a area change, and the interaction *q* value with precipitation (PRE) reaches 78.3%.

From the perspective of soil erosion rate, ELE∩SL, PD∩SL and ELE∩SL have the maximum interaction value of 83.5%, 85% and 82.1% in 2011, 2022 and 12 years. The dominant role of erosion rate change in the study area is the interaction between slope length and elevation, which is also affected by landscape patch density. The results show that the change of soil erosion rate in this area is caused by human activities in the natural environment, especially regional economic development, combined with landscape density and connectivity.

## 4. Discussion

### 4.1. Achievements in erosion control

Compared with 2011, the function of soil and water conservation in the region has been significantly improved, which is due to the implementation of ecological projects such as comprehensive management of small river basins as units, as well as the positive interaction between socio–economic development and soil and water conservation. Soil erosion is mainly affected by factors such as rainfall, topography and land use type. Among these factors, rainfall and land use types vary greatly. For example, woodland, as an important part of the natural environment, its vegetation canopy can intercept rainwater and its roots can preserve soil and sand, thus reducing soil erosion caused by rainfall in the region [43]. The mitigation of soil erosion in Luoning, Song and Yichuan is closely related to the comprehensive soil and water loss control projects continuously invested in sloping farmland. Taking Song County as an example, in 2021, 7000 acres of horizontal terraces were newly built in 6 administrative villages. 7 new drainage and irrigation projects, 7 storage ponds, 7 sand sinks and several field production roads, drains and signs were built. At the beginning of 2023, the treatment degree of Song County at the end of the period was more than 70%, and the retention rate of comprehensive treatment measures was higher than 80%. After 2023, it organized 4 administrative villages to carry out comprehensive soil and water loss control projects on sloping farmland. At present, the area of forest land in the region has increased, and the projects such as ditch head protection and runoff drainage have been gradually promoted. Therefore, the soil erosion in the region can be reduced by strengthening the sealing and protection of forest land.

At the same time, Luoyang city has carried out dynamic monitoring of soil erosion in key basins of in the southern mountainous areas, and executed comprehensive management of small watersheds centered on soil erosion prevention. These measures primarily encompass constructing silt dam systems and implementing slope land modification projects, with particular emphasis on water–harvesting and drainage infrastructure for sloping terrains, promoting water–efficient agriculture, retiring farmland for afforestation and grassland restoration, thereby rehabilitating and expanding vegetated areas in forest land and grass land.

### 4.2. Persistent challenges and policy recommendations

The slight increase in soil erosion on transportation and construction land is attributed to development pressures from the region’s abundant mineral and tourism resources, concentrated near Luoyang, a major industrial city in central China. The area of transportation land and construction land has increased. With the rapid economic growth, supporting measures for mineral exploitation and tourism also need to be gradually improved. There are 41 kinds of minerals with proven reserves in the study area, which are distributed in Luoyang, an important industrial city in central Henan Province. The development zone with concentrated mineral resources is prone to serious soil erosion, so it is necessary to strengthen the supervision and inspection of soil and water conservation in resource development to prevent new soil erosion caused by production and construction activities. The decline of erosion area in orchard is due to the rational planning of orchards in the region, the development of characteristic fruit forests, especially steep slope planting, requiring scientific selection of tree species, reasonable determination of planting scale, pay attention to the protection of the original surface vegetation and topsoil.

When combined with natural factors, the influence of landscape pattern in the study area significantly improved the interpretation ability of each landscape index to changes in soil erosion area and erosion rate [19]. Regional landscape pattern stability decreased, patch connectivity weakened, and heterogeneous landscape interaction near the boundary reduced regional soil and water conservation ability [44]. Human activities are one of the causes of landscape fragmentation. They will lead to the simplification and change of landscape structure [45–47], leading to the increase of landscape ecological risk. Previous studies have found that landscape pattern index is highly correlated with soil conservation [43,48]. For example, the NP and PD of traffic land increase, and the expansion and fragmentation are obvious, resulting in the fragmentation of the landscape and the expansion of the erosion area. The result showed the POP was the dominant factor for the change of soil erosion area between 12 years. The interactive effect of slope gradient and POP exerts significant influence on both soil erosion rates and soil erosion area. The food demand brought by urbanization and population growth in the study area is causing changes in land use function, and at the same time increasing the risk of soil erosion [49]. Land reclamation and construction projects in steep slope areas increased runoff and sediment yield on due to changes in land use driven by population growth.

### 4.3. Limitations of quantitative evaluation of soil erosion

Although widely used for evaluating interrill erosion and rill erosion, the CSLE model is generally not suitable for estimating gully erosion. Existing research indicates significant gully erosion within the study area, characterized by an average gully length of 902.55 m and a total gully coverage of 11,578.78 km^2^, representing 41.7% of its total area [6]. Constructing a comprehensive soil erosion evaluation system for this region based on the spatial distribution and dynamics of interrill erosion and gully erosion constitutes a key focus for future research. The physicochemical properties of soil have a significant impact on soil erosion. However, this study encountered difficulties in obtaining large–scale soil attribute data, which limited a comprehensive analysis of the factors influencing soil erosion.

## 5. Conclusion

A multi–stage, unequal probability, systematic area sampling method was used to determine the field investigation units in the loess hilly area of western Henan Province, and the soil erosion rates in the field investigation units were calculated by using the CSLE model to evaluate the soil erosion status in 2011 and 2022. On this basis, the spatiotemporal changes of soil erosion in the loess hilly area of western Henan Province in the past 12 years were analyzed, and the driving forces of soil erosion evolution were analyzed by using the geographical detector method. The research conclusions were as follows:

The soil erosion in the loess hilly area of western Henan showed a trend of “double decline”. The average soil erosion rate decreased by 25.5%, from 7.97 t·ha ⁻ ¹ in 2011 to 5.94 t·ha ⁻ ¹ in 2022. The percentage of soil erosion area in 2011 was 44.10%, which was reduced to 29.10% in 2022. In 2011, high soil erosion rates were concentrated in southeastern Yiyang County, southwestern Yichuan County, northern Song County, and southeastern Luoning County, areas which additionally corresponded to the regions with the highest density of eroded gullies.

Soil erosion mainly occurred in forest land and cultivated land, followed by construction land, orchard land and grassland. The altitude of soil erosion was mainly distributed at less than 200 m and 400–600 m. In 2011, soil erosion occurred in areas with gradients greater than 25°, followed by 15°–25° and 6°–15°. In 2022, the area above 25° have the largest percentage of erosion, followed by the slope area from 15° to 25°and below 6°.

In the loess hilly area of western Henan, the fragmentation degree of landscape pattern was intensified, the stability of landscape pattern was reduced, and the conservation ability of regional soil and water resources was decreased. In the study area, the number and density of all types of land patches, except orchard land, increased significantly from 2011 to 2022. Although the overall shape of patches in different regions was more regular, the boundary perimeter increased, the boundary shape became more tortuous, the segmentation degree of landscape pattern increased, the aggregation degree decreased, and the fragmentation degree increased.

The results of geographic detector showed that soil erosion was affected by landscape fragmentation and was the result of the interaction of multiple factors. The results of soil erosion area percentage factor detection showed that the dominant factors of soil erosion area in 2011 and 2022 were mainly slope and slope length, with *q* values of 45% and 28.8%, respectively, but the explanatory power decreased in 12 years. POP, with *q* value of 15.1%, was the dominant factor for the change of soil erosion area between 12 years. The diversity index enhanced the explanatory ability of soil erosion area, and the driving force of soil erosion area change gradually changed from socio–economic to landscape fragmentation. The dominant factor of soil erosion rates and its 12–year change was still the slope length factor, with explanatory power of more than 14%, which was basically consistent with the influencing factors of erosion area change (S1 Data).

## Supporting information

S1 Table Geodetector analysis results data. (XLSX)

S1 Data Soil erosion rates and percentage of soil erosion area. (ZIP)
